# Production of Organic Acids from Cashew Nut Shell Liquid (CNSL) via Electrochemical Synthesis

**DOI:** 10.3390/ijms262210821

**Published:** 2025-11-07

**Authors:** Jorge A. Ducuara, Alvaro A. Arrieta, Oriana Palma Calabokis

**Affiliations:** 1 Department of Biology and Chemistry, University of Sucre, Sincelejo 700001, Colombia; jorge_ducuara@unisucrevirtual.edu.co; 2 Faculty of Engineering and Basic Sciences, Fundación Universitaria Los Libertadores, Bogotá 111221, Colombia

**Keywords:** organic acids, chronopotentiometry, electrosynthesis, cashew nut shell liquid (CNSL), electrochemical oxidation

## Abstract

Environmental problems arising from conventional production models have posed a significant challenge in the search for renewable sources as raw materials for the production of everyday chemical compounds through more sustainable alternatives. The objective of the present work was the electrochemical synthesis of organic acids from the liquid of the natural and technical cashew nut shell (CNSLn and CNSLt), employing chronopotentiometry using a potentiostat and a graphite working electrode. Two concentrations (0.01–0.1% *v*/*v*) of CNSLn and CNSLt, two concentrations of NaOH as supporting electrolyte (0.125–2 M), and two current densities (40–60 mA/cm^2^) were tested in the experiments. Organic acids were detected and quantified by HPLC. To characterize the redox processes occurring in the constituents of CNSL, spectroelectrochemical analysis (FTIR–cyclic voltammetry), FTIR, and chronoamperometry were performed. The maximum concentrations obtained in the treatments were: acetic acid (828.86 mg/L), lactic acid (531.78 mg/L), and formic acid (305.4 mg/L), while other acids present in lower concentrations included oxalic, propionic, citric, and malonic acids. Voltammetry characterizations showed three irreversible oxidation processes in the anodic wave during the first cycle, indicating that the first process involved the formation of the phenoxy radical, the second process the formation of hydroquinones and benzoquinones, and the third process the cleavage of the aromatic ring and the aliphatic chain to form the organic acids. Furthermore, another oxidation pathway was observed, consisting of a fourth process in the second voltammetry cycle, corresponding to the nucleation of the phenoxy radical, evidenced as the formation of the C–O–C bond visible at 1050 cm^−1^ in the infrared spectrum. From this route, a polymer was formed on the electrode surface, which limited the yield of organic acid synthesis. Finally, this research provides new insights in the field of electrochemistry, specifically in the synthesis of organic acids from CNSL as a renewable feedstock, with the novelty being the production of oxalic, propionic, citric, and malonic acids.

## 1. Introduction

Pollution and the accumulation of waste from various industries have severely affected ecosystems, causing consequences that are sometimes irreversible to both biotic and abiotic components. These situations have resulted in direct and indirect repercussions on human health [1,2,3,4]. In response to the above, and in order to address this environmental problem caused by production models involving the use of non-renewable raw materials and production cycles with oxidizing and reducing agents, in recent decades, new sustainable production methods based on renewable and low-cost raw materials have been explored [5].

One renewable source that has been widely studied is cashew nut shell liquid (CNSL), due to its alkylphenolic composition, whose properties have been exploited for applications in various industrial, agricultural, medicinal, and other sectors [6,7,8,9,10,11]. Anacardic acid, cardanol, and cardol are the constituents of CNSL, and their proportions vary depending on the CNSL extraction method [12].

CNSL is referred to as natural CNSL (CNSLn) when extraction does not involve temperatures above 180 °C, and the proportions of its constituents are: anacardic acid 60–65%, cardanol 10%, cardol 15–20%, and traces of 2-methylcardol. On the other hand, it is called technical CNSL (CNSLt) when extraction involves temperatures between 180–200 °C, generally consisting of cardanol 60–65%, cardol 15–20%, anacardic acid 0–2%, 2-methylcardol 1.2–4.1%, and traces of polymeric material [13,14].

In addition to studies linking a wide variety of applications of CNSL and its constituents, recent electrochemical research has obtained high-value-added organic acids by applying electrochemical oxidation to effluents containing CNSLt, suggesting that phenolic constituents can be cleaved to synthesize acetic acid, oxalic acid, malonic acid, and other acids [15,16].

Although effluents from the cashew industry have been treated using electrochemical techniques, with research focusing on the use of BDD, DSA, or Ti/RuO_2_IrO_2_TiO_2_ anodes [15,16,17], there is also the possibility of exploring the direct electrochemical transformation of CNSLn and CNSLt using more accessible electrodes such as graphite electrodes, aiming to contribute new knowledge in this field, specifically oriented toward renewable, sustainable, and technically viable production.

Moreover, in Colombia, the cashew agroindustry sector shows an annual production growth of 7% [18]. This has also led to an increase in discarded waste such as shells and/or effluents, generating environmental concerns, as CNSL is a pollutant whose lethal doses and concentrations have already been studied in mammals and aquatic fauna [19,20]. Additionally, in rural areas of the departments of Sucre and Córdoba, this agroindustry depends on seasonal harvesting, and the waste generated from the shelling process receives no treatment or valorization as an alternative source of income due to limited access to technological alternatives.

Based on the ideas presented, this research focuses on the transformation of CNSLn and CNSLt through electrochemical techniques as a conversion and characterization mechanism, testing conditions that allow the study of performance patterns, thereby contributing to the utilization and reduction of this type of waste, to the renewable production of organic acids, and to the advancement of this field in the long term as an alternative that could impact rural economies by diversifying their income through science, technology, and innovation.

## 2. Results and Discussion

**Figure 1:**
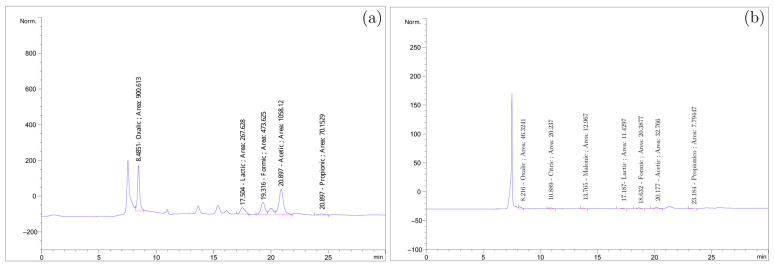
(**a**) HPLC experiment 1; (**b**) HPLC experiment 32.

The electrochemical experiment using chronopotentiometry resulted in the synthesis of the following organic acids: citric acid, propionic acid, malonic acid, oxalic acid, lactic acid, formic acid, and acetic acid, which were detected and quantified by HPLC (Figure 1).

Acetic, formic, and oxalic acids have already been reported from the treatment of effluents using CNSLt as a raw material in Medeiros et al. [15,16]. It is important to note that the electrochemical treatment of CNSLn and CNSLt had not been previously reported, and the novelty of this work lies in the synthesis of propionic, malonic, lactic, and citric acids from CNSL.

The concentrations of these organic acids varied according to specific experimental conditions. Therefore, conditions that yielded a high-performance pattern for the respective organic compounds were selected. These yields were calculated based on the initial mass of CNSL. It is worth highlighting that lactic acid, formic acid, and acetic acid showed the highest synthesis concentrations, while the other detected organic acids were produced in lower concentrations, as shown in Table 1.

The results suggest that there are specific conditions that directly affect the production of the main organic acids, with notable yields found for acetic acid (828.86 mg/L; 82.9%), lactic acid (531.78 mg/L; 53.2%), and formic acid (305.24 mg/L; 30.5%), while yields for the other organic acids did not exceed 11%, calculating the maximums for citric acid (33.88 mg/L; 3.39%), propionic acid (109.65 mg/L; 10.97%), malonic acid (17.46 mg/L; 1.75%) and oxalic acid (106.85 mg/L; 10.69%), For these organic acids that were obtained in lower concentrations, further studies should analyze the possible optimization for their synthesis. The results showed the selectivity of citric acid at lower concentrations of CNSLn (0.01% *v*/*v*), NaOH (0.125 M) and 40 mA/cm^2^, propionic acid obtained its highest concentration when using CNSLt (0.1% *v*/*v*), NaOH (0.125 M) and 40 mA/cm^2^, malonic acid was selective to CNSLt (0.1% *v*/*v*), Na_2_SO_4_ (0.125 M) and 40 mA/cm^2^, and oxalic acid obtained its maximum concentration when applying CNSLt (0.1% *v*/*v*), Na_2_SO_4_ (2 M) and 60 mA/cm^2^, these synthesis values show a different behavior to the predominant organic acids that were mostly selective to the use of high concentrations of NaOH (2 M).

Based on these results, a simplified table was created showing the conditions that led to the most optimal production yields (Table 2).

The results establish a relationship between the type and concentration of CNSL, the electrolyte concentration, and the applied current density for the production of organic acids. In the case of CNSLt, the maximum concentrations of acetic acid were reached at 2 M electrolyte concentration, 0.1% *v*/*v* CNSL, and 60 mA/cm^2^ current density, similarly to CNSLn under the same conditions, with CNSLt showing more promise for this organic acid.

Regarding formic acid, the highest concentrations were obtained with CNSLn, suggesting a probable selectivity of the compound depending on the type of CNSL used. For lactic acid, the maximum concentrations were achieved with CNSLt, particularly when the current density was lower (40 mA/cm^2^).

The results suggest that increasing the NaOH concentration enhances the production of organic acids. This relationship arises because the increase in electrolyte concentration not only improves the system’s conductivity but also dissolves the CNSL [15]. As the electrolyte concentration increases, a greater proportion of CNSL molecules are dissolved and thus become susceptible to oxidation.

An increase in current density can promote a higher oxidation rate on the electrode surface and simultaneously a greater cleavage of the constituents [15,21], although undesired reactions such as polymerization may occur, compromising the synthesis yield of organic acids [22].

**Figure 2:**
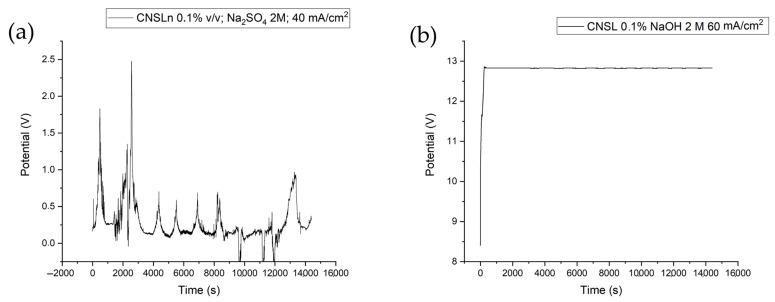
(**a**) Chronopotentiogram of experiments: NaOH 0.125 M, CNSLn 0.1% *v*/*v*, 60 mA/cm^2^; (**b**) NaOH 2 M, CNSLn 0.1% *v*/*v*, 60 mA/cm^2^.

In the chronopotentiograms (Figure 2), the potential behavior under different experimental conditions was observed. In most cases, voltage fluctuations characterized by an increase and decrease in electrode resistance are evident; this phenomenon is attributed to the delayed electron transfer kinetics at the surface. At this point, polymeric layers form as coatings that occupy active reaction sites, consequently leading to low organic acid production yields [23,24].

However, under the condition of CNSLt 0.1%, NaOH 2 M, and a current density of 60 mA/cm^2^, a stable behavior is observed, and it is precisely under this condition that the highest acetic acid production is expressed, with a yield value of 82.9%.

### 2.1. Spectroelectrochemical Analysis

**Figure 3:**
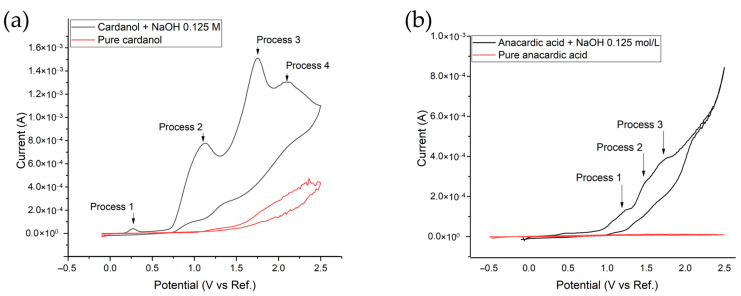
(**a**) Cyclic voltammetry of cardanol (**b**) anacardic acid at one cycle.

Cyclic voltammetry showed that the constituents of CNSL are not conductive by themselves at pure concentrations, requiring the addition of a supporting electrolyte (NaOH 0.125 M) to carry out the process (Figure 3). The CNSL constituents exhibited a common behavior characterized by the presence of three irreversible oxidation processes and the formation of a loop in the anodic wave (Figure 3).

**Figure 4:**
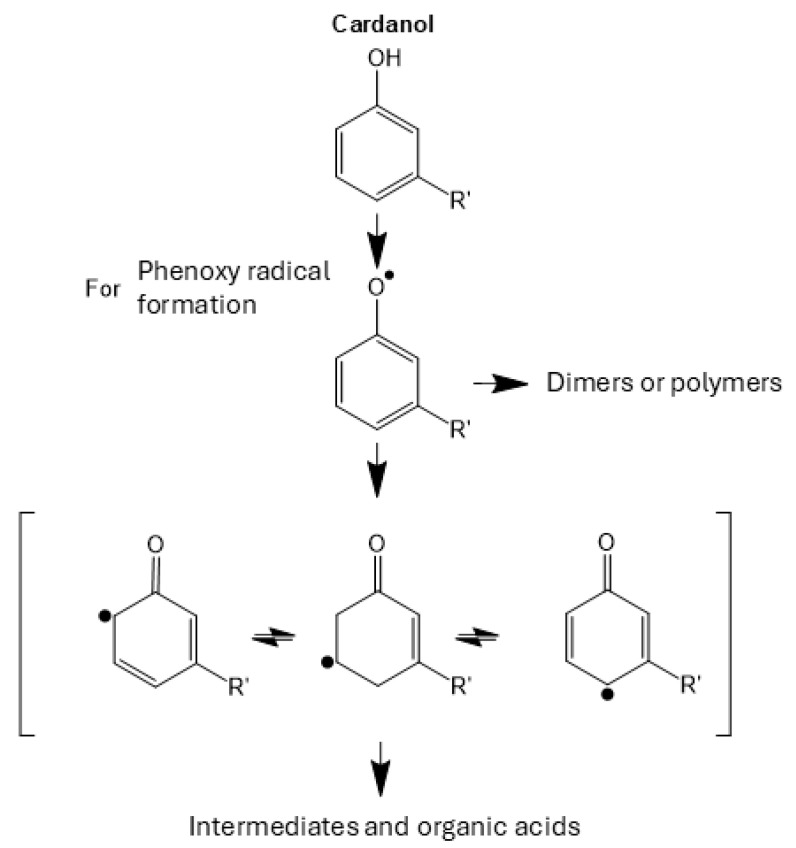
Oxidation processes of cardanol during the first cycle.

These oxidation processes govern the transformations of cardanol and anacardic acid during electrosynthesis. The first process corresponds to the formation of the phenoxy radical, the second to the formation of hydroquinones and benzoquinones, and in the third oxidation process, the aromatic ring opens, giving rise to hydrocarbon chains that are subsequently cleaved to form organic acids [16,25]. These processes are schematically illustrated below (Figure 4).

The oxidation could be evidenced by a change in the infrared spectrum of cardanol, also observed in anacardic acid by Arrieta et al., 2025 [25]. The first oxidative peak appears as a low-intensity peak in the region around 1050 cm^−1^ (Figure 4), indicative of the presence of carboxylates (C–O–C) [26,27]. This corresponds with the loop observed in the previous voltammogram and suggests nucleation of the phenoxy radical. This oxidation pathway leads to the formation of dimers or polymers.

**Figure 5:**
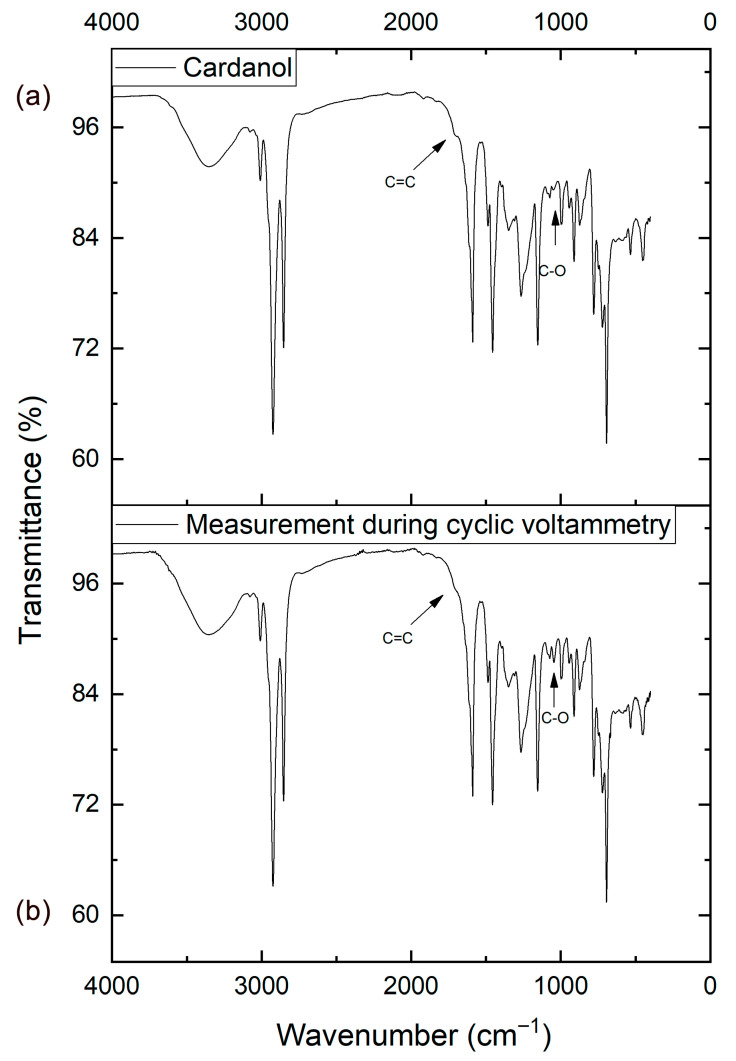
(**a**) Spectrum of cardanol and during cyclic voltammetry (**b**).

The second oxidative process manifests as a change in the region near 1698 cm^−1^, associated with C=C vibrations of the aromatic ring. As the reaction proceeds, the intensity of the C=C bond peak decreases, suggesting the formation of intermediates such as ethyl derivatives and aliphatic hydrocarbons produced by the cleavage of the aromatic ring (Figure 5).

**Figure 6:**
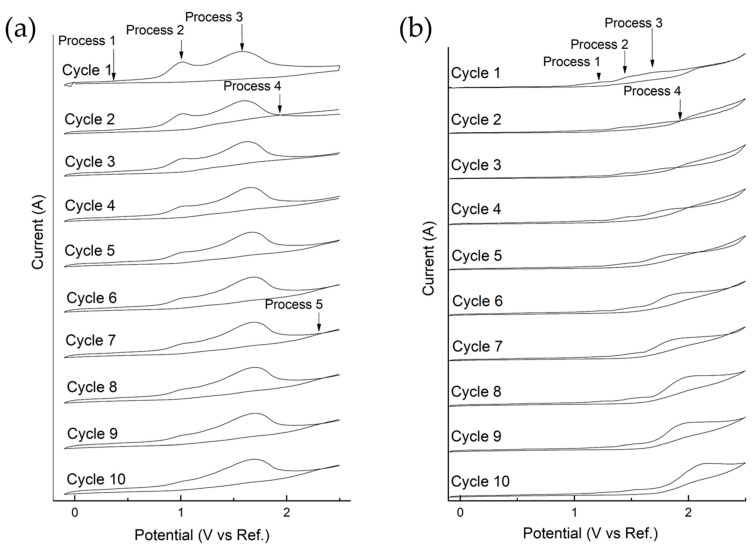
(**a**) Cyclic Voltammetry—Cardanol, (**b**) Anacardic Acid at 20 mV/s and 10 scan cycles.

On the other hand, the phenomena observed in the spectrum and voltammogram allowed a more detailed study of the oxidation processes of the constituents by applying 10 scan cycles at 20 mV/s. The results of this characterization are shown for both constituents, displaying similar behavior (Figure 6).

Referencing the study on anacardic acid by Arrieta et al., 2025 [25], and in this case for cardanol, three irreversible oxidation processes are observed in the anodic wave during the first cycle. For cardanol, the first process occurs at 0.4 V, leading to the formation of the phenoxy group by the loss of hydrogen from the phenol’s OH group. Subsequently, the second oxidation process occurs at 1.01 V, producing hydroquinones and benzoquinones, and the third oxidation process, occurring at 1.57 V, involves the cleavage of intermediates to form low molecular weight organic acids [16,25,28], it should be noted that organic acids can react with the alcohols formed in the electrosynthesis process, forming ethyl and methyl esters, which react with OH radicals, causing the cleavage of the esters into organic acids, CO_2_, and H_2_O. Oxidative processes in general for this type of compound mainly generate carboxylic acids [16,29].

In the second cycle, a loop forms at 1.94 V, which corresponds to the nucleation of phenoxy radicals [30]. These radicals undergo oligomerization or polymerization, evidenced by the coating formed on the electrode surface. It can be observed how the nucleation loop shifts toward more positive potentials in subsequent cycles, disappearing by the fifth cycle at 2.47 V (in anacardic acid, it disappears by the sixth cycle).

The shift in nucleation possibly indicates progressive polymerization and changes in the electrode’s surface properties, which affect the nucleation potential and the yield of the electrochemical synthesis. Again, in the seventh cycle, a new nucleation appears, which could be related to phenoxy radicals that have not yet been consumed, forming polymeric material once more.

**Figure 7:**
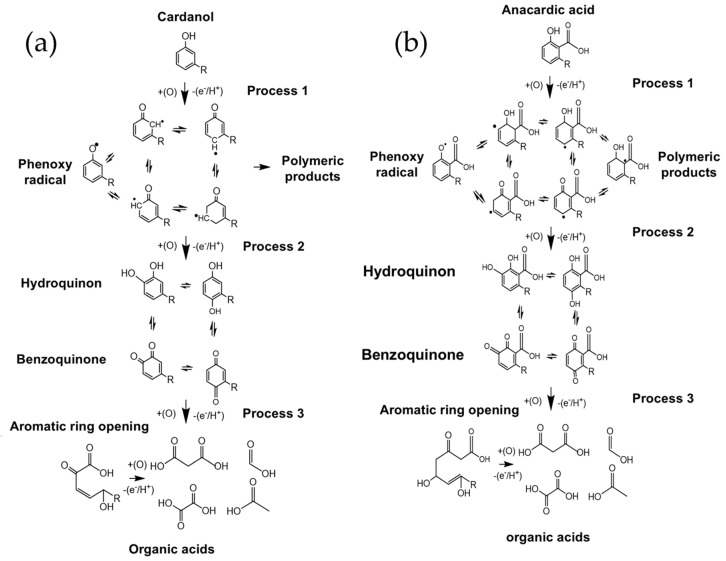
Scheme of the oxidation reactions of (**a**) cardanol and (**b**) anacardic acid, into low molecular weight organic acids and polymeric material.

Based on the voltammogram studies, these phenomena were expressed in a diagram representing the reactions occurring during the electrochemical oxidation process of CNSL constituents (Figure 7), applying the cyclic voltammetry potentials described above. Some studies have proposed that the formed oligomers or polymers can undergo oxidations that degrade them into CO_2_ and H_2_O [16]. However, in several conducted experiments, no polymer degradation was observed. At the end of the electrooxidation process, for CNSLn, the polymer appeared as a copper-colored crust, while for CNSLt, the polymers were characterized by a thin coating on the electrode surface.

These electropolymerization processes have been studied previously, with polymerization proceeding through the phenoxy radical via consecutive addition reactions [31,32].

To verify that the constituents decompose into organic acids as shown in the schemes (Figure 7), electrochemical synthesis was carried out using anacardic acid and cardanol, the major constituents in CNSLn and CNSLt, respectively. The experiment was performed by chronopotentiometry under conditions similar to the CNSL treatment, as outlined in Table 3.

### 2.2. Infrared Spectroscopy Characterization

**Figure 8:**
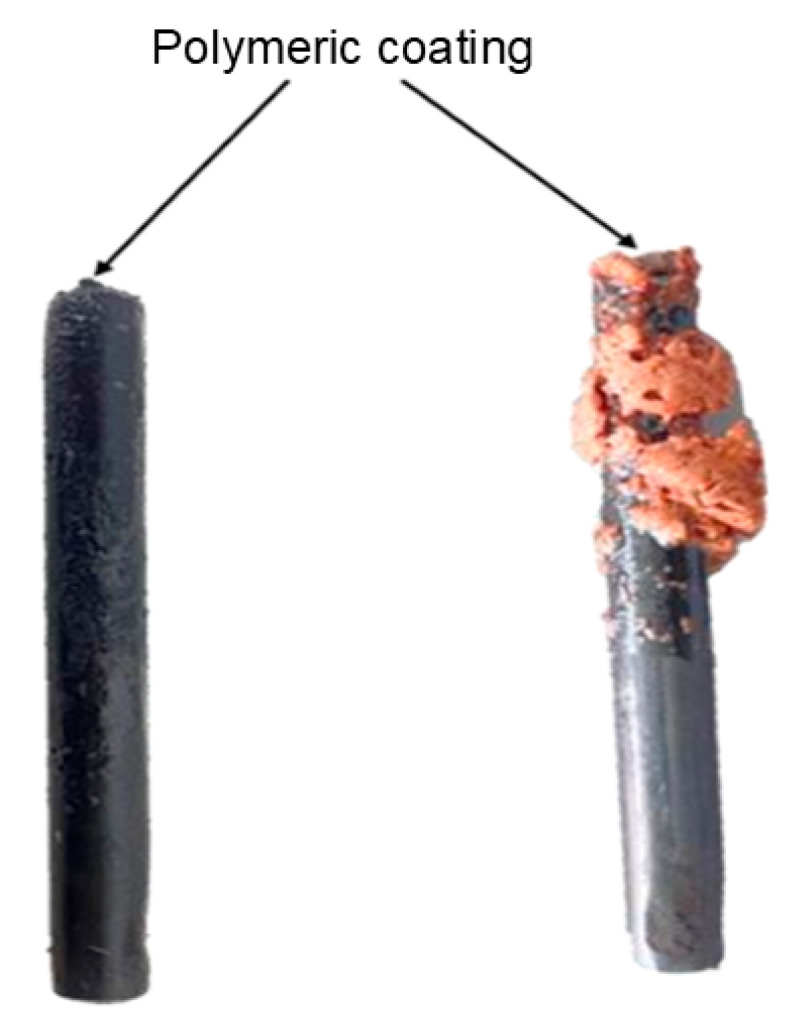
Polymeric material as a coating on: cardanol (**left**) and anacardic acid (**right**).

The characterized material corresponded to the polymeric layers formed on the working electrode surface. It was observed that in experiments with CNSLt, these layers appeared as a thin, dark-colored coating, unlike the experiments with CNSLn, which exhibited a copper-colored crust (Figure 8).

**Figure 9:**
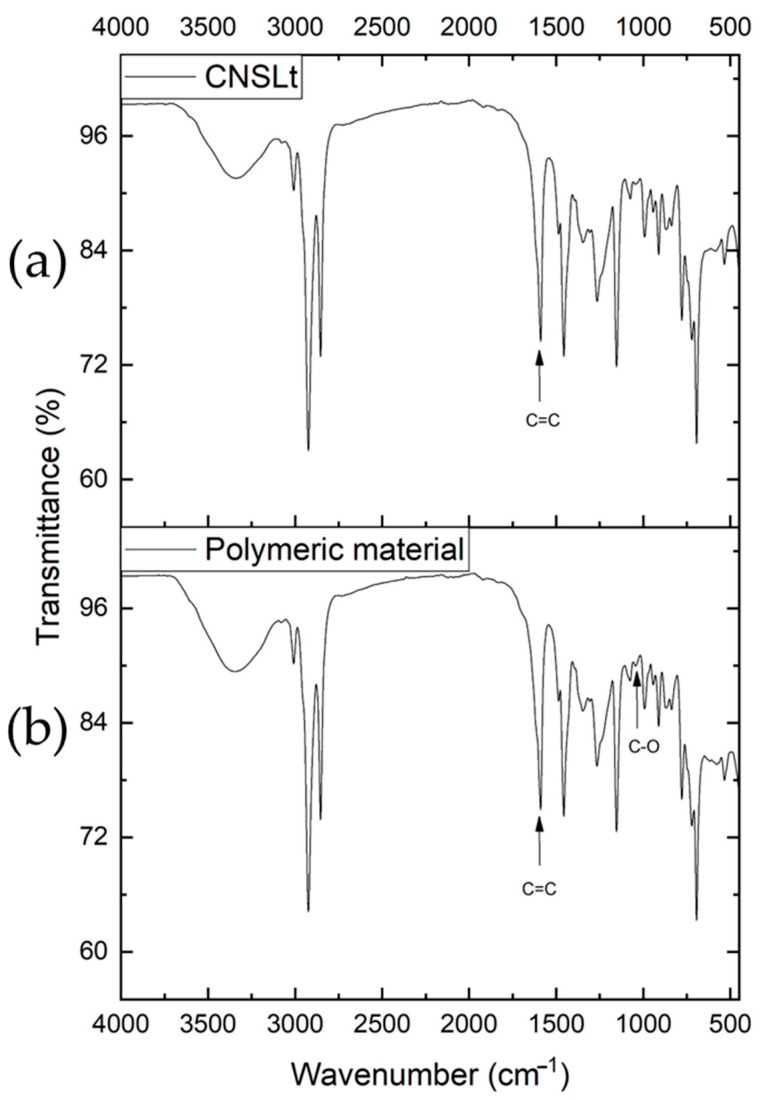
Infrared spectrum: (**a**) cardanol; (**b**) polymeric material.

The polymeric material derived from CNSLt that coats the electrode was characterized by a dark color. Infrared measurements revealed peaks corresponding to CNSLt, highlighting a peak in the 1040 cm^−1^ region attributed to the C–O group (Figure 9), which is the nucleation point for the formation of this type of polymer through the oxidation pathways previously described [25,31,32].

**Figure 10:**
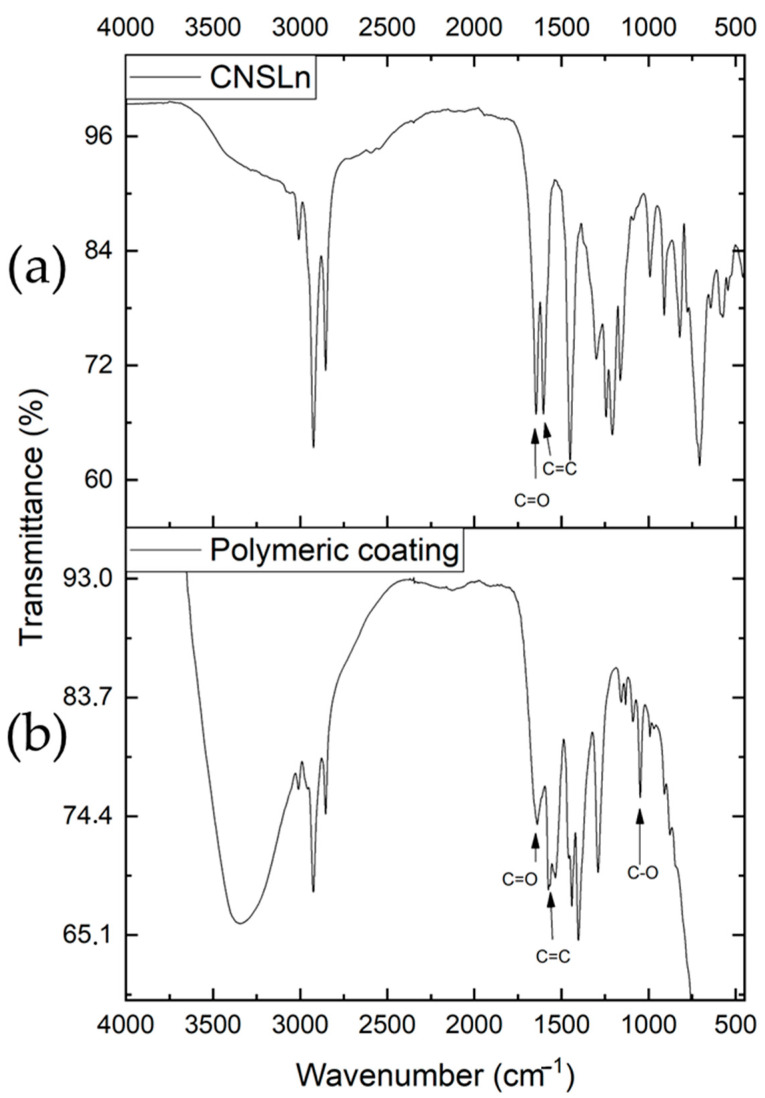
Infrared spectrum: (**a**) anacardic acid; (**b**) polymeric coating.

On the other hand, the polymeric material derived from CNSLn in the infrared spectrum shows the functional groups of anacardic acid (Figure 10). It also exhibits a peak in the 1046 cm^−1^ region corresponding to the C–O bond. These types of polymers pose limitations for the formation of organic acids, but they may also represent a research field regarding insulating coatings and their properties.

The electropolymerization of these phenols is influenced by various factors such as monomer concentration, electrolysis parameters, electrode material, and electrolyte type, among others [32,33]. In this study, the conditions listed in Table 2 suggest they are optimal to minimize the formation of such coatings on the electrode surface. This conclusion is supported by the yields reported earlier (Table 1 and Table 2).

It is important to highlight the use of the graphite electrode, whose porous surface favors the cleavage reactions of organic matter. This characteristic facilitates the diffusion and transport of electrolytes and electroactive species, while providing a greater number of active sites for reactant interaction and reaction, allowing the simultaneous transformation of organic substances [34]. The high efficiency of the electrode makes it a sustainable resource due to its renewable origin and low acquisition cost, making it an accessible material. Additionally, the established conditions appear promising for scalability and the development of technology based on CNSL as a renewable raw material through electrochemical techniques.

## 3. Materials and Methods

### 3.1. Reagents

Natural CNSL (CNSLn) was extracted from cashew nut shells by pressing under ambient temperature and pressure conditions. The cashew nut shell used was from Anacardium occidentale Yucao Ao3 variety provided by the company ASOPROMARSAB (Chinú, Cordoba, Colombia). Technical CNSL (CNSLt) was obtained by thermal treatment of CNSLn at 180–200 °C for 4 h. For electrochemical experiments, the following reagents were used: sodium hydroxide (NaOH; Sigma Aldrich, St. Louis, MO, USA; 99.9%), sodium sulfate (Na_2_SO_4_; Sigma Aldrich, St. Louis, MO, USA, 99.9%), ethanol (C_2_H_6_O; Merck, Darmstadt, Germany; 99.8%), methanol (CH_3_OH; Panreac Química S.L.U., Barcelona, Spain; 99.9%), ammonium hydroxide solution (NH_3_; Sigma Aldrich, St. Louis, MO, USA, 28–30%), hexane (CH_3_(CH_2_)_4_CH_3_; Merck, Darmstadt, Germany), hydrochloric acid (HCl; Panreac Química S.L.U., Barcelona, Spain; 37%), Celite 545 (Merck, Darmstadt, Germany), calcium hydroxide (Ca(OH)_2_; Merck, Darmstadt, Germany; 99.9%), ethyl acetate (C_4_H_8_O_2_; Merck, Darmstadt, Germany; 99.8%), and activated carbon.

Cardanol was obtained by liquid–liquid extraction, which consisted of dissolving 50 g of CNSLt in methanol (160 mL) and adding ammonium hydroxide (25%, 100 mL), stirring for 15 min. Extraction was carried out with hexane (4 × 100 mL). The extract was then washed with 5% HCl (50 mL) and subsequently with distilled water (50 mL). Activated carbon (5 g) was added, stirred for 10 min, and filtered through Celite (7.5 g). The filtrate was dried over anhydrous sodium sulfate and concentrated to obtain pure cardanol [13].

Anacardic acid was obtained by dissolving 50 g of CNSLn in 200 mL of 5% methanol, then slowly adding 25 g of calcium hydroxide until a precipitated salt (calcium anacardate) was formed. The salt was filtered, washed with distilled water, heated at 45–50 °C for 3 h, and then suspended in a solution of distilled water and 11 M HCl. The acid was then extracted and dried over 17.5 g of anhydrous sodium sulfate [12,13].

### 3.2. Electrochemical Synthesis of High-Value-Added Organic Acids

The main organic acids synthesized by electrosynthesis are summarized in Table 4, which presents the experiment consisted of mixing CNSLt or CNSLn (0.01% *v*/*v*, 0.1% *v*/*v*) in an electrolyte solution composed of 25% ethanol and NaOH (0.125–2 M). A saturated calomel reference electrode, a graphite working electrode, and a platinum counter electrode were connected to a Gamry Interface 1010E potentiostat (Gamry Instruments, Warminster, PA, USA) and immersed in the prepared solution. The applied current densities were 40 mA/cm^2^ and 60 mA/cm^2^. The electrochemical experiment lasted approximately 4 h, and the technique employed was chronopotentiometry [15].

### 3.3. Detection of Organic Acids by HPLC

Characterization and quantification were performed using an Agilent 1100 Series High-Performance Liquid Chromatography (HPLC) system with a UV/Vis detector and a SUPELCOGEL H column for organic acid detection (Agilent Inc., Santa Clara, CA, USA). The operating conditions were as follows: Injection volume: 20 μL; Flow rate: 0.5 mL/min; Mobile phase: 0.1% H_3_PO_4_ solution.

### 3.4. Spectroelectrochemical Analysis

This technique was performed by cyclic voltammetry at 10 mV/s using a DropSens 110 screen-printed carbon electrode (Metrohm, Asturias, Spain), with cardanol mixed with NaOH and 25% ethanol in a 4:1 ratio. Simultaneously with cyclic voltammetry, infrared spectra were recorded using a PerkinElmer Spectrum Two IR spectrometer (PerkinElmer Inc., Shelton, CT, USA).

### 3.5. Cyclic Voltammetry Characterization

Cyclic voltammetry was carried out using a DropSens 110 screen-printed carbon electrode (Metrohm, Asturias, Spain) immersed in a solution of 25% ethanol, 0.125 M NaOH, and anacardic acid or cardanol at a concentration of 0.1% *v*/*v*. The voltammetry conditions were as follows: Initial potential: −0.5 V; Final potential: 2.5 V; Scan rate: 20 mV/s; Number of cycles: 10.

## 4. Conclusions

This study establishes a solid foundation for the development of scalable electrochemical technologies. The efficient and economically competitive production of value-added compounds from cashew nutshell liquid (CNSL) residues depends on the optimization and precise control of CNSL and NaOH concentrations, as well as current density. This technology has the potential to drive industrial advances toward the production of organic acids through a clean, renewable, cost-effective, and innovative model, with a positive impact on the environment and on the diversification of local economies that face technological limitations. In this study, CNSLn showed yields for acetic, formic, and lactic acids, but was more selective for formic acid production, while CNSLt proved more promising for lactic and acetic acid. The organic acids synthesized in lower concentrations are of particular interest for investigating specific conditions that could optimize their production. Thanks to the alkylphenolic structure of CNSL, it is possible to synthesize these compounds via electrochemical oxidation, successfully cleaving its structure into organic acids. This research contributes new knowledge on sustainable applications of CNSL. Finally, it is recommended to consider economic feasibility studies.

## Figures and Tables

**Table 1:** Organic acids synthesized by electrosynthesis.

CNSL-n	CNSL-t	Electroly Salts (M)		Current Density	Organic Acids Concentration (mg/L)						
% *v*/*v*	% *v*/*v*	Na_2_SO_4_	NaOH	mA cm^−2^	Citric Acid	Propionic Acid	Malonic Acid	Oxalic Acid	Lactic Acid	Formic Acid	Acetic Acid
0.01	-	0.125	-	40	9.12	10.02	6.79	1.83	33.1	11.52	38.72
0.01	-	2	-		17	-	10.65	-	39.73	19.84	281.96
0.1	-	0.125	-		12.19	-	-	0.46	-	9.1	199.78
0.1	-	2	-		-	-	8.81	58.69	29.59	21.9	28.77
0.01	-	-	0.125		33.88	-	3.92	10.81	60.22	58.94	121.78
0.01	-	-	2		12.85	-	-	21.59	269.48	58.83	132.64
0.1	-	-	0.125		-	13.5	-	45.58	41.44	34.64	65.99
0.1	-	-	2		-	-	-	65.93	327.25	189.09	378.1
-	0.01	0.125	-		-	11.3	11.14	1.4	31.51	9.98	89.98
-	0.01	2	-		17.16	-	9.01	-	35.2	12.28	26.99
-	0.1	0.125	-		11.46	-	17.46	-	13.57	15.47	189.41
-	0.1	2	-		-	-	8.32	62.59	-	12.41	567
-	0.01	-	0.125		-	38.83	-	-	19.32	58.96	133.09
-	0.01	-	2		13.98	-	15.25	13.88	250.57	156.57	431.79
-	0.1	-	0.125		-	109.65	-	41.42	371.68	140.29	248.88
-	0.1	-	2		-	-	-	66.42	531.78	261.7	573.92
0.01	-	0.125	-	-	-	7.6	106.85	1.15	29.1	8.71	77.3
0.01	-	2	-		14.35	-	12.8	-	34.39	16.37	167.61
0.1	-	0.125	-		10.83	-	-	1.85	5.43	14.38	205.38
0.1	-	2	-						-	13.13	37.68
0.01	-	-	0.125		-	34.19	10.37	9.01	49.97	51.44	154.77
0.01	-	-	2		-	16.17	-	18.03	395.16	266.13	572.09
0.1	-	-	0.125		-	68.23	-	49.03	58.16	60.99	123.54
0.1	-	-	2		-	-	-	55.46	405.88	305.24	744.3
-	0.01	0.125	-		-	-	11.94	1.63	35.77	8.79	17.7
-	0.01	2	-		12.51	-	9.73	7.13	37.4	10.46	21.4
-	0.1	0.125	-		-	-	-	1.05	-	5.8	21.71
-	0.1	2	-		-	-	-	-	-	9.07	17.44
-	0.01	-	0.125				6.73	6	38.45	16.4	37.14
-	0.01	-	2		-	17.15	-	20.24	399.25	75.45	99.69
-	0.1	-	0.125		-	20.76	8.12	13.08	80.99	48.83	71.43
-	0.1	-	2		-	60.55	-	37.35	264.71	220.59	828.86

**Table 2:** Production yields of acetic, formic, and lactic acids.

CNSL % *v*/*v*		NaOH (M)	Current Density (mA/cm^2^)	Acetic Acid (mg/L)	Formic Acid (mg/L)	Lactic Acid (mg/L)	Yield (%)
CNSLt	CNSLn						
0.1	-	2	60	828.86	-	-	82.9
-	0.1	2	60	744.3	-	-	74.4
0.1	-	2	60	-	220.59	-	22.1
-	0.1	2	60	-	305.24	-	30.5
0.1	-	2	40	-	-	531.78	53.2
-	0.1	2	60	-	-	405.88	40.6

**Table 3:** Synthesis of organic acids from cardanol and anacardic acid.

Organic Substrate		Electrolyte		Organic Acids (mg/L)							
Anacardic Acid (% *v*/*v*)	Cardanol (% *v*/*v*)	Na_2_SO_4_ (M)	NaOH (M)	Citric Acid	Propionic Acid	Malonic Acid	Tartaric Acid	Oxalic Acid	Lactic Acid	Formic Acid	Acetic Acid
0.1			0.125			11.8		9.27		18.45	85.72
	0.1		0.125		103.69			48.43		132.25	898.74

**Table 4:** Experimental conditions for the electrosynthesis of organic acids from natural and technical CNSL.

Experiment	CNSL-n	CNSL-t	Electrolyte Salts (M)		Current Density	Electrolysis Time
#	% *v*/*v*	% *v*/*v*	Na_2_SO_4_	NaOH	mA/cm^−2^	Min
1	0.01	-	0.125	-	40	240
2	0.01	-	2	-		
3	0.1	-	0.125	-		
4	0.1	-	2	-		
5	0.01	-	-	0.125		
6	0.01	-	-	2		
7	0.1	-	-	0.125		
8	0.1	-	-	2		
9	-	0.01	0.125	-		
10	-	0.01	2	-		
11	-	0.1	0.125	-		
12	-	0.1	2	-		
13	-	0.01	-	0.125		
14	-	0.01	-	2		
15	-	0.1	-	0.125		
16	-	0.1	-	2		
17	0.01	-	0.125	-	60	
18	0.01	-	2	-		
19	0.1	-	0.125	-		
20	0.1	-	2	-		
21	0.01	-	-	0.125		
22	0.01	-	-	2		
23	0.1	-	-	0.125		
24	0.1	-	-	2		
25	-	0.01	0.125	-		
26	-	0.01	2	-		
27	-	0.1	0.125	-		
28	-	0.1	2	-		
29	-	0.01	-	0.125		
30	-	0.01	-	2		
31	-	0.1	-	0.125		
32	-	0.1	-	2		

## Data Availability

The original contributions presented in this study are included in the article. Further inquiries can be directed to the corresponding author.
